# Chronic Kidney Disease Impairs Bone Defect Healing in Rats

**DOI:** 10.1038/srep23041

**Published:** 2016-03-09

**Authors:** Weiqing Liu, Ning Kang, Dutmanee Seriwatanachai, Yuliang Dong, Liyan Zhou, Yunfeng Lin, Ling Ye, Xing Liang, Quan Yuan

**Affiliations:** 1 State Key Laboratory of Oral Diseases, West China Hospital of Stomatology, Sichuan University, Chengdu, China; 2 Department of Oral Biology, Faculty of Dentistry, Mahidol University, Bangkok, Thailand

## Abstract

Chronic kidney disease (CKD) has been regarded as a risk for bone health. The aim of this study was to evaluate the effect of CKD on bone defect repair in rats. Uremia was induced by subtotal renal ablation, and serum levels of BUN and PTH were significantly elevated four weeks after the second renal surgery. Calvarial defects of 5-mm diameter were created and implanted with or without deproteinized bovine bone mineral (DBBM). Micro-CT and histological analyses consistently revealed a decreased newly regenerated bone volume for CKD rats after 4 and 8 weeks. In addition, 1.4-mm-diameter cortical bone defects were established in the distal end of femora and filled with gelatin sponge. CKD rats exhibited significantly lower values of regenerated bone and bone mineral density (BMD) within the cortical gap after 2 and 4 weeks. Moreover, histomorphometric analysis showed an increase in both osteoblast number (N.Ob/B.Pm) and osteoclast number (N.Oc/B.Pm) in CKD groups due to hyperparathyroidism. Notably, collagen maturation was delayed in CKD rats as verified by Masson’s Trichrome staining. These data indicate that declined renal function negatively affects bone regeneration in both calvarial and femoral defects.

Chronic kidney disease (CKD) has clearly been considered a major public health concern according to the National Kidney Foundation^1^,^2^. In the United States, its prevalence is 13.1%, affecting 26.3 million people^3^. Dwarfing this figure, an estimated 119.5 million Chinese people (10.8%) have some stage of CKD^4^. Moreover, the incidence and prevalence of CKD are expected to increase markedly over the coming decades in conjunction with aging, obesity and a rising incidence of diabetes and hypertension.

Declined renal function is often complicated with phosphocalcic metabolic disorders, which subsequently impact bone structural integrity^5^ and eventually lead to mineral and bone disorders (CKD-MBD)^6^. CKD has been increasingly recognized as a risk factor for osteoporosis and fragility fractures in humans^7^. Patients with end-stage renal disease (ESRD) are at considerably higher risk for vertebral fracture^8^ and hip fracture^9^, which increase while kidney function declines^10^. Clinically, patients on dialysis exhibited significant mineralized bone loss characterized by generalized thinning of cortical bone^11^, and patients with predialysis CKD and fractures have lower areal bone mineral density (aBMD) according to dual-energy x-ray absorptiometry and lower volumetric BMD (vBMD), thinner cortices, and trabecular loss according to HR-pQCT^12^. Furthermore, Melissa *et al.* reported a significant reduction in cortical mandibular bone thickness in a CKD mouse model^13^.

Dental implantation has become the ideal method for restoring missing teeth. CKD is one of the most commonly encountered relative contraindications for dental implant therapy^14^. With the prevalence of CKD dramatically increasing worldwide^1^,^3^,^4^,^15^,^16^,^17^, the number of the individuals with missing teeth who will require dental implants is expected to grow. Thus, the effectiveness of tissue engineering approaches for regenerating bone in bone defects needs to be determined before considering application to CKD patients.

In dental practice, the most widely used bone substitute is deproteinized bovine bone mineral (DBBM), which has favorable experimental and clinical documentation and relatively long follow-up^18^,^19^,^20^. Recently, a composite of spongious DBBM along with 10% porcine collagen fibers in block form was introduced to achieve a better adaptation to the defect site^21^,^22^,^23^. In contrast, Gelatamp, which is made of 95% foam gelatin sponge and 5% finely dispersed colloidal silver, is routinely used to pack the surgical site to prevent infection and facilitate wound healing^24^,^25^.

Our previous study revealed that the bone/implant contact ratio and strength of bone-implant integration were significantly lower in the CKD group at 2-week healing, which indicated that CKD negatively affects an early fixation of titanium implants in mice^26^,^27^. A large body of epidemiological and experimental evidence has also shown that renal failure will inevitably lead to declined bone health^28^,^29^, and CKD might impact the healing of bone fractures^30^. However, the effect of CKD on the regeneration of bone defects is largely unknown.

In this study, we generated CKD rats by 5/6 renal ablation and then studied their bone regeneration using both critical-sized calvarial bone defects and femoral cortical bone defects. Our data indicate that declined renal function negatively affects bone regeneration.

## Materials and Methods

### Ethics Statement

This study was conducted in strict accordance with the international standards stated in the Guide for the Care and Use of Laboratory Animals of the National Institutes of Health and the ARRIVE guidelines (http://www.nc3rs.org/ARRIVE). All the experiments performed were approved by the Subcommittee on Research and Animal Care (SRAC) of Sichuan University. All surgery was performed under anesthesia by intraperitoneal injection of a combination of ketamine (100 mg/kg) and xylazine (10 mg/kg), and in addition, buprenorphine (0.05 mg/kg) was given for perioperative analgesia to minimize suffering and pain.

### Animals

Three-month-old male SD rats (body weight: 300~350 g) were obtained from the Experimental Animal Center of Sichuan University and randomly assigned to CKD and sham groups. The animals were kept under climate-controlled conditions (25 °C; 55% humidity; 12 hours of light alternating with 12 hours of darkness) and fed a standard diet.

### Surgical procedure to induce uremia

**Figure 1:**
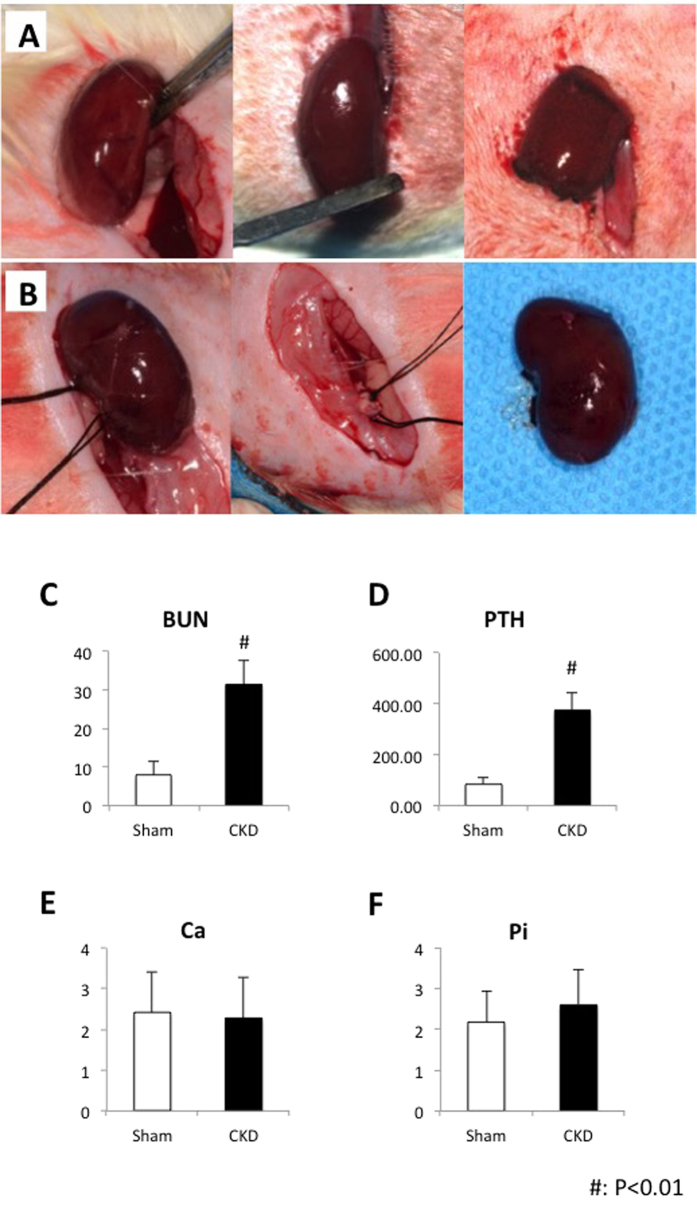
CKD rat model established by a two-step 5/6 nephrectomy. (**A**) First renal surgery involves electrocautery of the left kidney except for a 2-mm area around the hilum. (**B**) Second renal surgery was performed one week later by double ligation of the renal hilum with silk suture and surgical excision of the right kidney. Serum biochemical measurements. (**C**) serum BUN; (**D**) serum PTH; (**E**) serum calcium; (**F**) serum phosphate. ^#^*p* < 0.01.

CKD was induced by a two-step 5/6 nephrectomy as described previously^26^. Briefly, the first renal surgery involved electrocautery of the left kidney except for a 2-mm area around the hilum (Fig. 1A). A second renal surgery was performed one week later by double ligation of the renal hilum with silk suture and surgical excision of the right kidney (Fig. 1B). Sham surgery consisted of anesthetic, flank incision exposing the kidney and closure of the abdominal wall.

### Serum Biochemical Assays

Four weeks after the second renal surgery, blood was collected by cheek pouch puncture. Serum biochemistry was performed using the following commercially available kits: blood urea nitrogen (BUN) (Roche Diagnostics, Indianapolis, IN, USA); PTH (Immutopics, San Clemente, CA, USA); and Calcium and Phosphate (Stanbio Laboratory, Boerne, TX, USA), following the manufacturer’s protocols.

### Bone Defect Surgeries

**Figure 2:**
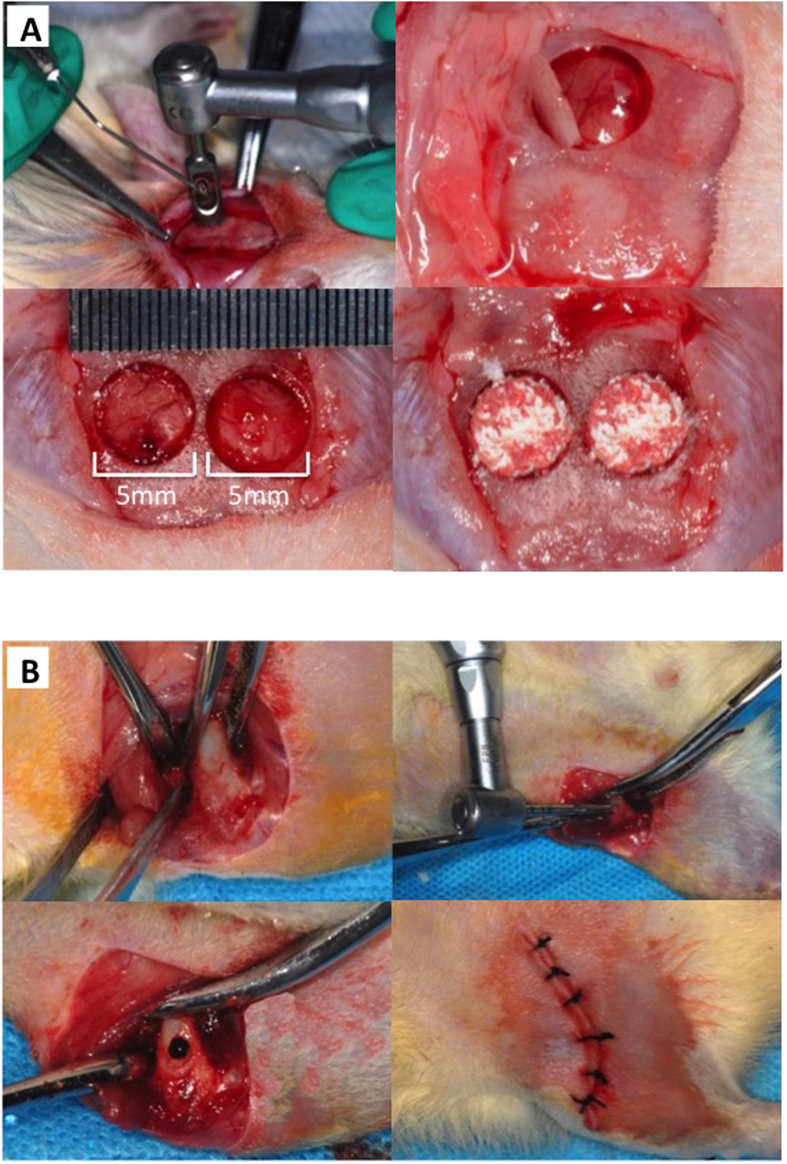
Brief surgical procedures to create bone defects. (**A**) Two symmetrical full-thickness critical-sized (diameter: 5 mm) calvarial bone defects implanted with or without DBBM. (**B**) Through-and-through cortical femoral bone defect (diameter: 1.4 mm) in the anterior-distal surface.

After establishment of CKD, the rats were subjected to bone defect surgeries either in the calvaria (Fig. 2A) or in femurs (Fig. 2B).

Critical-sized defects (CSDs) were created in the cranium following the surgical protocol published by Patrick *et al.*^31^. After careful exposure of the flat surface of the cranium via skin incision, two 5.0-mm symmetrical full-thickness bone defects were created across the sagittal suture using a trephine drill (Stoma^®^, Germany). The defects were implanted with deproteinized bovine bone mineral (DBBM, Bio-Oss^®^ Collagen, Geistlich, Wolhusen, Switzerland) or left empty to serve as control. To ensure consolidation of the implant material, the periosteum was closed using interrupted 5-0 Monocryl sutures before skin closure. Animals were sacrificed at 4 and 8 weeks post-operatively with an overdose of ketamine.

For femoral cortical bone defects, the anterior-distal surfaces of the femurs were exposed by blunt dissection of the quadriceps after skin incision. Through-and-through perforations (1.4 mm) that disrupted cortex, periosteal and endosteal surfaces were then generated using a round bur (Komet^®^, Germany) operating at 10,000 rpm under ample irrigation with saline. After thoroughly rinsing to remove bone fragments, gelatin sponge (Gelatamp) was implanted into the defects to stabilize the blood clot. In the light of a previous study regarding cortical defect healing in normal mice^32^, there was hematoma formation at day 3, new bone formation at day 7 and day 10, and the defect region underwent remodeling at day 14 and day 21. Therefore, the rats were euthanized on week 2 and 4 post-injury.

Calvarial and femoral specimens were then fixed in 4% paraformaldehyde for 48 h at 4 °C before transferring into 70% ethanol and stored at 4 °C.

### X-Ray Microtomography

Samples were scanned in a Skyscan 1176 micro-CT imaging system (Skyscan, Kontich, Belgium) at a spatial resolution of 18 μm (X-ray source 50 kV/455 kA; exposure time 0.265 sec; magnification 15×; 1 mm filter applied). Volumetric reconstructions and analyses were performed using built-in software NRecon 1.6 and CTAn 1.8, respectively. Thresholding of gray values was performed using the histogram tool to separate mineralized elements from background. Finally, 3D images of the samples were reconstructed using CTvox.

For the analysis of calvarial defect healing, the volume of interest (VOI) was defined as a cylindrical area covering the initial bone defect. Then, bone volume/total volume (BV/TV, %) was calculated within the delimited VOI.

For femoral cortical analysis, VOI was a 1.4-mm-diameter, 0.3-mm-thick cylinder positioned between the edges of the defect. Bone volume/total volume (BV/TV, %) and bone mineral density (BMD, mg/cm^3^) were determined.

### Histological analysis

After micro-CT scanning, the fixed samples were soaked in 17% ethylenediamine tetraacetic acid (EDTA) at pH 7.3 with daily solution replacement until complete decalcification. Then, the specimens were rinsed in PBS, dehydrated with ascending graded alcohol, cleared in xylene and embedded in paraffin. Initiating from the center of the original surgical defect, serial 5-μm-thick sections were cut perpendicularly to the sagittal suture or along the femoral shaft axis. Decalcified paraffin sections were stained with hematoxylin and eosin (H&E), tartrate-resistant acid phosphatase (TRAP) and Masson’s Trichrome (MT) for light microscopy observation (Nikon Eclipse 80i) to investigate bone formation, resorption and maturation, respectively. Images were then captured using NIS-Elements software.

### Histomorphometric analysis

Three histological sections representing the central area of the original surgical defect were selected for histological and histomorphometric analysis. Images of new bone formation were digitized and histomorphometrically analyzed with NIH ImageJ software (National Institutes of Health, USA). New bone formation in the femoral cortical gap was quantified as the ratio of the area of newly formed bone to that of total defect area.

Osteoblast and osteoclast enumeration was performed in the femoral cortical gap as described previously^33^. The number of osteoblast and osteoclast cells was averaged and signified the osteoblast number per bone surface (N.Ob/B.Pm, mm^−1^) and osteoclast number per bone surface (N.Oc/B.Pm, mm^−1^), respectively.

### Statistical analysis

All values were presented as the mean ± SD. Statistically significant differences were assessed by an independent student *t* test for comparison between two groups. A *p* value of less than 0.05 was considered to be statistically significant.

## Results

### Confirmation of CKD animal model prior to bone defect surgery

Serum BUN (Fig. 1C) increased by approximately 4-fold in the CKD group compared with the sham-operated group, indicating a successful establishment of the uremic rat model. Serum PTH (Fig. 1D) levels, which correspond to the degree of hyperparathyroidism, were elevated by 3.4-fold in the CKD group, further confirming impaired renal function. However, serum calcium (Fig. 1E) and phosphate (Fig. 1F) levels were not altered between CKD rats and sham controls.

### General postsurgical observation of the rats

All rats tolerated the surgical procedures well, and survived throughout the study. After the femoral cortical bone defect surgeries, no mortality or femoral fractures were recorded. All rats were able to walk normally at 18 h after surgery. No infection or exposure of the wound was observed after the surgery.

### CKD impairs calvarial critical-sized bone defect healing

**Figure 3:**
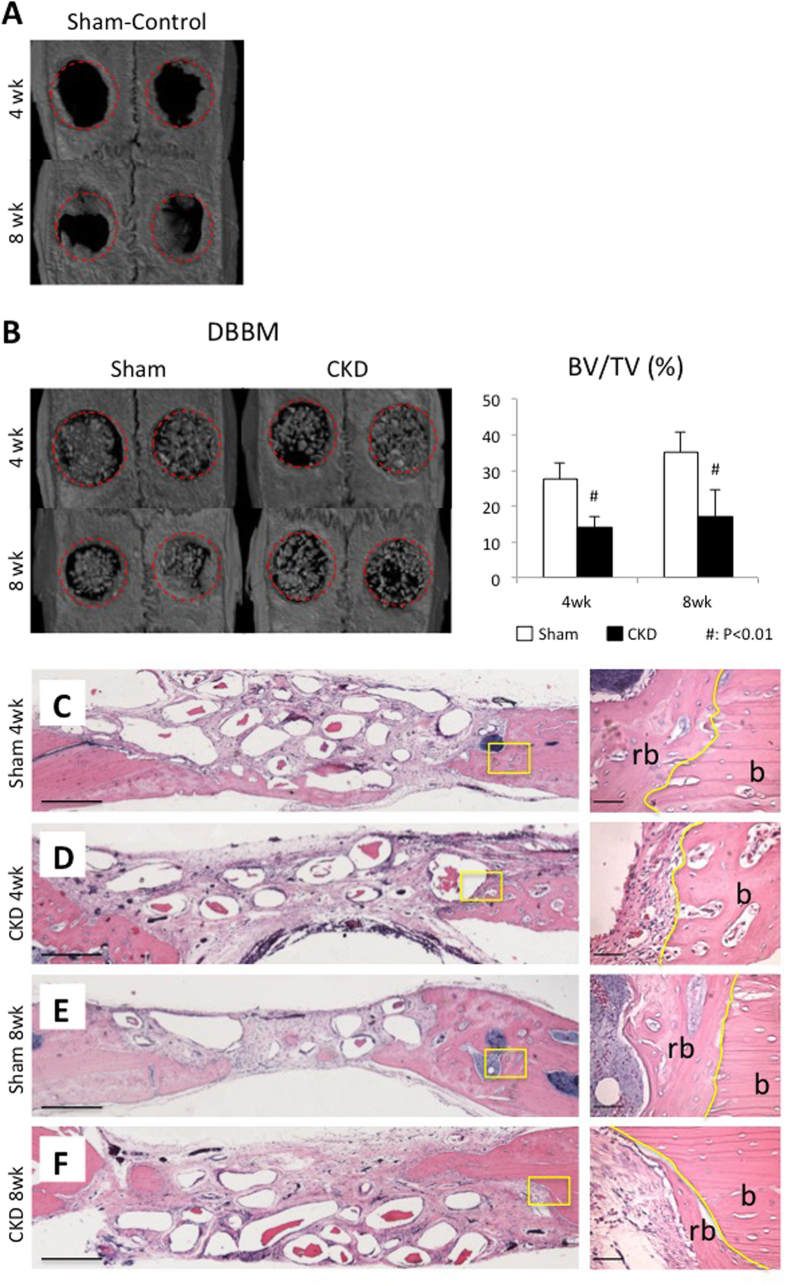
Impaired calvarial critical-sized bone defect healing in CKD rats. Micro-CT analysis: representative 3D reconstruction images and quantitative morphometric analysis of bone volume/total volume (BV/TV, %) within the original defect area of 4-week specimens (**A**) and 8-week specimens (**B**). Red-dotted lines refer to the ROI (region of interest). n = 10, ^#^*p* < 0.01. (**C–F**) Histological analysis: representative HE–stained histological images of all DBBM-implanted groups. High magnification (scale bar = 50 μm) of the solid-line rectangle in the left panoramic images (scale bar = 500 μm). b, host bone; rb, regenerated bone.

As shown in the representative 3D reconstructed μCT images of the empty control groups, *de novo* bone formation was almost absent at the 4-week healing interval (Fig. 3A). Even after 8 weeks of healing, the untreated defects remained largely open with limited bone regeneration only at the defect margin (Fig. 3B). This finding confirmed that a 5-mm-diameter parietal intra-osseous defect in rats cannot be healed over the experimental period; therefore, it fulfills the requirements for what has been defined as a “critical size defect” (CSD).

For defects implanted with DBBM, the threshold of gray values was performed to separate remnant particles from newly regenerated bone tissue (Fig. 3B). The BV/TV was significantly lower in the CKD group compared with the sham group at week 4 (13.99 ± 3.13 vs 27.54 ± 4.41,49.2% reduction, *p* = 0.002) and week 8 (17.17 ± 7.36 vs 35.19 ± 5.49, 51.2% reduction, *p* = 0.00066). Taken together, the capacity to regenerate new bone by CKD rats is approximately only half of that of sham controls for both the untreated empty defects and DBBM-implanted defects.

Consistent with the radiographic findings, histological observation also showed decreased volume of newly regenerated bone in CKD rats (Fig. 3C–F). After 8 weeks of healing, there was modest regenerated bone at the defect margin in the sham group (Fig. 3C). There was much less newly regenerated bone in CKD mice (Fig. 3E,F). Notably, there is a visible gap between the regenerated bone (rb) and original bone (b) in the CKD group, which also suggests impaired bone repair (Fig. 3F).

### CKD impairs femoral cortical bone healing

**Figure 4:**
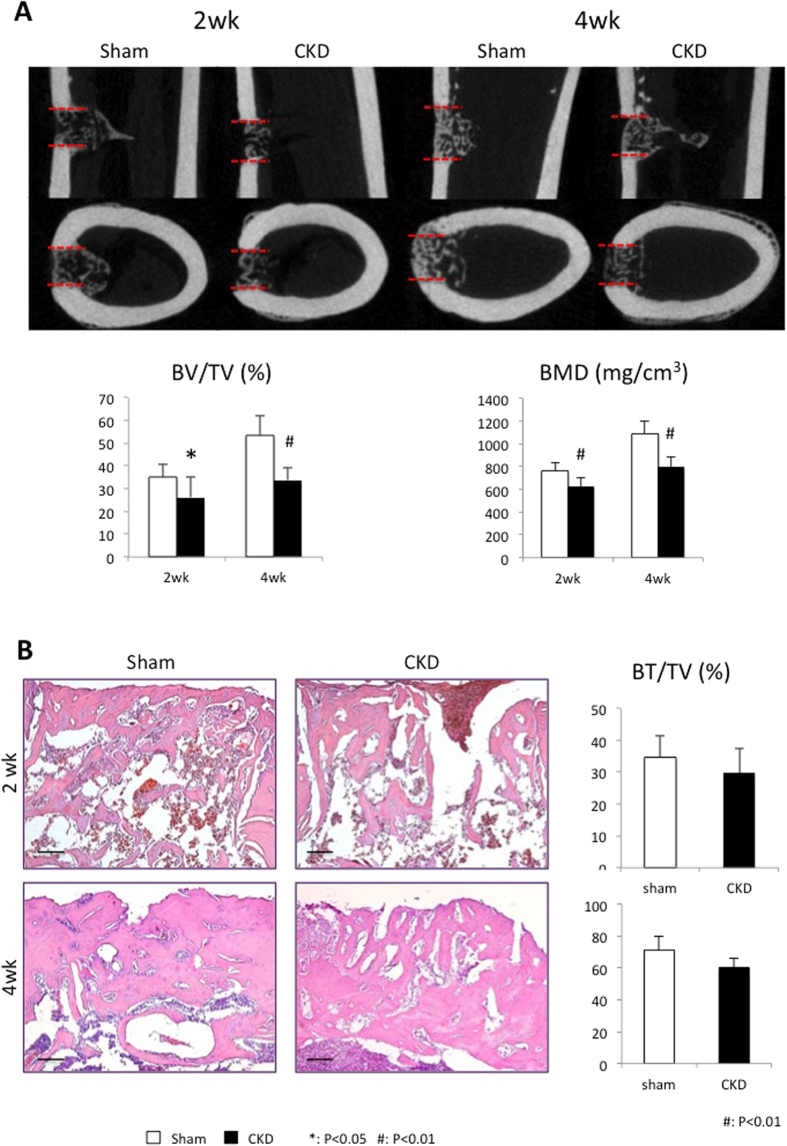
Impaired femoral cortical bone defect healing in CKD rats. (**A**) Micro-CT analysis: representative 2D coronal and axial images of each group. Red-dotted lines delineate the position of the original defect margin. Quantitative morphometric analysis of bone volume/total volume (BV/TV, %) and bone mineral density (BMD, mg/cm^3^) in the cortical gap. n = 10, ^#^*p* < 0.01. (**B**) Histological analysis: representative HE–stained histological images of the defect area. Quantification of new bone formation ratio (%) within the cortical gap. Scale bar = 500 μm.

We next evaluated bone regeneration in a femoral cortical bone defect model. Micro-CT analysis illustrated that a modestly mineralized callus partially bridged the defect region at the 2-week healing interval, and at week 4, the cortical gap was completely bridged (Fig. 4A). However, the volume of the mineralized callus (BV/TV, %) and extent of callus mineralization (BMD, mg/cm^3^) were significantly lower in the CKD group than in the sham group at both the 2- and 4-week healing interval. Bone healing capacity was reduced by approximately 20% in terms of new bone volume (2 week: 28.72 ± 5.56 vs 35.19 ± 5.78, *p* = 0.0017; 4 week: 58.82 ± 10.43 vs 71.05 ± 11.89, *p* = 0.00067). Callus mineralization was diminished by 18% at week 2 (623.62 ± 81.83 vs 758.62 ± 79.59, *p* = 0.000059) and 26.7% at week 4 (796.88 ± 88.07 vs 1087.84 ± 114.4, *p* = 0.00018).

Similar results were found in histological analysis (Fig. 4B). At week 2, the cortical gap was partially filled with woven bone, and the volume in CKD rats was significantly lower than that of control (34.68 ± 6.57 vs 29.86 ± 7.39). In addition, the spicules of woven bone were thicker and denser in sham rats compared with CKD rats. Interestingly, ingrowth of fibrous connective tissue into the defect area was observed in the CKD group, which might be attributed to the delayed formation of new bone tissue. At week 4, woven bone had been remodeled into mature lamellar bone in the sham group, whereas immature woven bone persisted in CKD rats.

**Figure 5:**
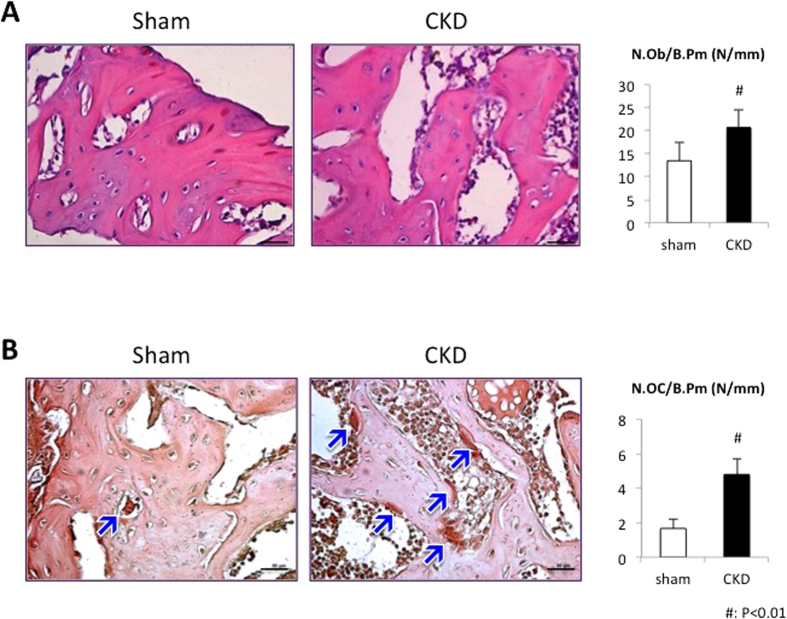
Histomorphometric analysis of newly regenerated bone in femoral cortical bone defects. (**A**) HE staining. The number of osteoblasts was averaged and signified the osteoblast number per bone surface (N.Ob/B.Pm, mm^−1^). (**B**) TRAP staining. Arrows indicate TRAP-positive cells. The number of osteoclasts was averaged and signified the osteoclast number per bone surface (N.Oc/B.Pm, mm^−1^). Scale bar = 50 μm.

We then performed histomorphometric analysis and found a 1.5-fold increase in osteoblast number per bone surface (N.Ob/B.Pm) in the CKD group (Fig. 5A). More importantly, the number of multi-nucleated TRAP-positive osteoclast cells (N.Oc/B.Pm) was also significantly elevated (as indicated by blue arrows) (Fig. 5B).

**Figure 6:**
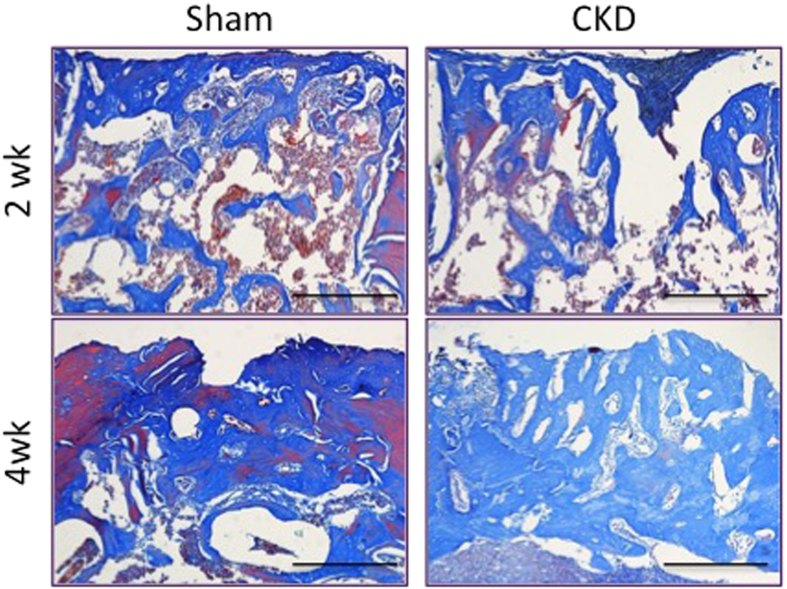
Delayed collagen maturation in CKD rats. Masson Trichrome staining of femoral defect area. Scale bar = 500 μm.

To investigate collagen maturation during the healing process, we then performed Masson’s Trichrome staining, with the mature bone matrix stained red and immature new bone matrix stained blue. While there is no noticeable difference in collagen maturation between sham and CKD at the 2-week healing interval, this contrast became visible at week 4 (Fig. 6). Furthermore, the defective region in the CKD group was largely stained blue, whereas there was red-stained mature collagen in the center of the cortical gap in the sham group, which indicates that collagen maturation was delayed in CKD rats.

## Discussion

It is widely accepted that CKD is associated with compromised bone health. To the best of our knowledge, the current study analyzes for the first time the impact of progressive CKD on bone defect healing *in vivo*. We found that declined renal function led to lower bone regeneration capacity in both the calvarial and femoral bone defect models.

The validity of the uremic model used in the present study was confirmed by serum biochemical changes. Four weeks after renal ablation, the CKD group exhibited markedly elevated serum BUN, suggesting that a bone defect was created when renal failure was already present. Substantiating the efficacy of the nephrectomy surgery, other biochemical markers of the uremic condition were also in line with previous studies published by other authors using similar CKD rat models^34^. Therefore, this *in vivo* animal model was suitable to imitate the metabolic disorders in CKD patients.

Calvarial critical-sized defects (5 mm in diameter) were established on the calvaria of CKD and sham-operated animals. This defect model has been widely used for assessment of the bone regeneration process^35^. In this context, CSDs have already been effectively used to report diminished bone regeneration capability in rodent models of diabetes^36^,^37^ and osteoporosis^38^, but no report has been published regarding its use in a CKD rat model.

Because untreated defects were shown to achieve minimal bone regeneration only at the margin after 8 weeks of healing in our pilot study, deproteinized bovine bone mineral (DBBM), which possesses excellent biocompatibility and osteoconductivity and is routinely used during guided bone regeneration (GBR), was implanted in subsequent experiments. The significant increase in BV/TV with DBBM implantation verified it as a competent bone grafting material even under renal failure conditions.

Micro-CT and histological findings consistently indicated an impaired bone defect healing process in CKD rats. However, BV/TV was not significantly different between sham and CKD groups for unfilled defects. This is very likely because without any bone grafting material, bone regeneration was too limited.

The original bone in CKD rats showed increased porosity at week 4, indicating that CKD rats were going through an active bone remodeling process. These changes in bone were indicative of excessive and uncontrolled hyperparathyroidism, which is also consistent with bone abnormalities found in the tibia of a CKD-MBD rat model^39^.

A bilateral cortical perforation of 1.4 mm in diameter was surgically created in the femur. This model neither necessitated a stabilization device nor disturbed the normal locomotion of the animal under our observation. Histological observation evidenced a little cartilage accumulation within the cortical gap at any time point in both CKD and sham rats. Unlike most other mechanical-instable fracture models^40^, this defect did not trigger a spread of cartilage tissue nor initiate an endochondral bone formation process, which is in agreement with previous studies on mice^32^,^41^,^42^,^43^ and rats^44^. Accordingly, the femoral bone defect healing model is characterized by dominant intramembranous ossification.

When compared with the calvarial defect model, the femoral defect model demonstrated more robust bone healing capacity. This could be because the bone marrow cavity in the femur could provide a prominent source of mesenchymal cells for the defect site, while bone marrow space is very limited in calvaria, and progenitor cells could be only derived from the defect margin and periosteum. This may explain the visible gap between new bone and original calvarial bone in histological sections in the CKD group.

Histomorphometric analysis of the femoral defects showed a 1.5-fold increase in osteoblast number along with a threefold increase in osteoclast number in CKD rats. These changes are most likely due to secondary hyperparathyroidism. There has been substantial evidence that continuous infusion of PTH decreases bone mass by stimulating a net increase in bone resorption^45^,^46^. Ma YL *et al.*^47^ reported that continuous infusion of PTH resulted in a corresponding dose-dependent increase in osteoclast number in parathyroidectomized (PTX) rats. On the other hand, markedly elevated PTH in systemic circulation increases osteoblast number by directly activating survival signaling in osteoblasts, as shown by previous *in vitro* and *in vivo* studies^48^,^49^. This delay of osteoblast apoptosis could partly explain the 1.5-fold increase in osteoblast number in our experiment.

In summary, to elucidate the influence of CKD on bone defect healing, we generated a CKD rat model by a two-step subtotal nephrectomy and then surgically created bone defects in calvaria or femurs. Micro-CT and histological analyses revealed a compromised newly regenerated bone volume with increased osteoblasts and osteoclasts and delayed collagen maturation. These data indicated that declined renal function impairs bone defect regeneration.

## Additional Information

**How to cite this article**: Liu, W. *et al.* Chronic Kidney Disease Impairs Bone Defect Healing in Rats. *Sci. Rep.* **6**, 23041; doi: 10.1038/srep23041 (2016).
