# Author Correction: Chronic Kidney Disease Impairs Bone Defect Healing in Rats

**DOI:** 10.1038/s41598-020-65651-4

**Published:** 2020-06-08

**Authors:** Weiqing Liu, Ning Kang, Dutmanee Seriwatanachai, Yuliang Dong, Liyan Zhou, Yunfeng Lin, Ling Ye, Xing Liang, Quan Yuan

**Affiliations:** 1 State Key Laboratory of Oral Diseases, West China Hospital of Stomatology, Sichuan University, Chengdu, China; 2 Department of Oral Biology, Faculty of Dentistry, Mahidol University, Bangkok, Thailand

Correction to: *Scientific Reports* 10.1038/srep23041, published online 09 March 2016

This Article contains errors. Figure 2A, which is an illustration of the surgical procedure, was inadvertently duplicated from a different publication provided below as Reference 1. Both studies followed the same surgical protocol, but implanted different biomaterials in the defects. The correct Figure 2A appears below as Figure 1.

**Figure 1:**
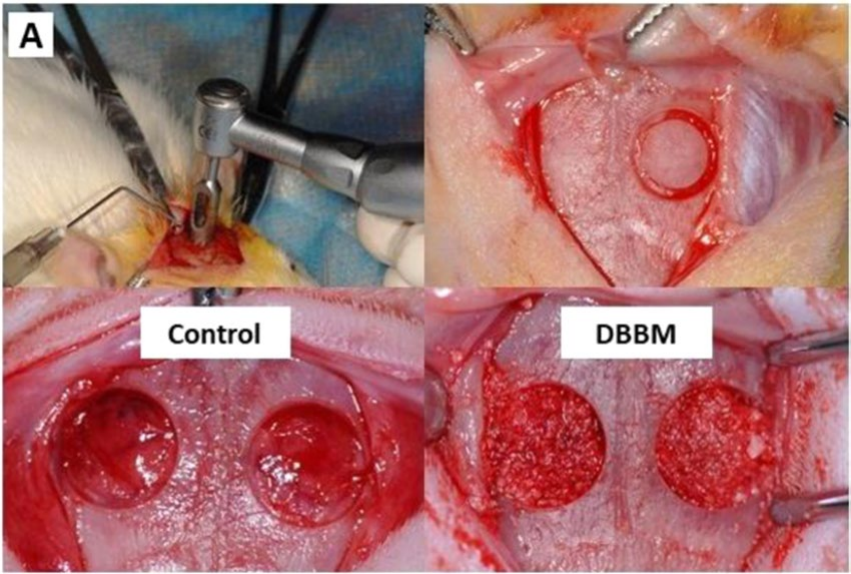
.

In addition, the bar graph in Figure 4A was inadvertently copied from the preliminary pilot study. The correct bar graph and raw data appear below as Figure 2 and Table 1, respectively.

**Figure 2:**
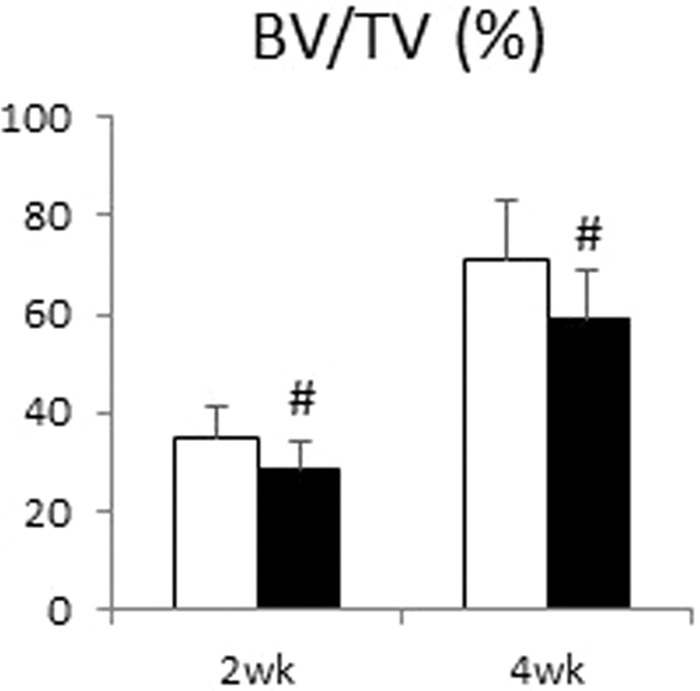
.

**Table 1:** .

BV/TV	sham 2wk	CKD 2wk	sham 4wk	CKD 4wk
1	36.29	28.15	62.15	48.44
2	28.87	22.71	83.88	64.57
3	38.08	36.86	61.15	57.25
4	43.17	25.86	88.47	69.69
5	28.35	24.33	62.17	52.10
6	39.35	25.91	71.08	45.07
7	39.15	22.06	82.67	71.59
8	27.17	35.47	79.55	45.30
9	31.10	35.66	66.14	66.47
10	40.35	30.23	53.25	67.74
